# Effects of perioperative dexmedetomidine on delayed graft function following renal transplant: a systematic review and meta-analysis

**DOI:** 10.1016/j.bjane.2024.844534

**Published:** 2024-07-02

**Authors:** Ka Ting Ng, Wei En Lim, Wan Yi Teoh, Soo Kun Lim, Ahmad Nazran bin Fadzli, Pui San Loh

**Affiliations:** a University of Malaya, Department of Anaesthesiology, Kuala Lumpur, Malaysia; b University of Glasgow, Department of Anaesthesiology, Glasgow, United Kingdom; c University of Malaya, Department of Medicine, Kuala Lumpur, Malaysia; d University of Malaya, Department of Surgery, Kuala Lumpur, Malaysia

**Keywords:** Dexmedetomidine, Kidney transplantation, Analgesia, Adrenergic alpha-2 receptor agonists, Pain, Glomerular filtration rate

## Abstract

**Background:**

Dexmedetomidine, a highly selective alpha-2 adrenoceptor agonist with sedative and analgesic effects, has been suggested in recent studies to possess renoprotective properties. Dexmedetomidine may reduce the incidence of delayed graft function and contribute to effective pain control post-renal transplantation. The primary objective of this systematic review was to assess whether dexmedetomidine decreases the occurrence of delayed graft function in renal transplant patients.

**Methods:**

Databases including MEDLINE, EMBASE, and CENTRAL were comprehensively searched from their inception until March 2023. The inclusion criteria covered all Randomized Clinical Trials (RCTs) and observational studies comparing dexmedetomidine to control in adult patients undergoing renal transplant surgery. Exclusions comprised case series and case reports.

**Results:**

Ten RCTs involving a total of 1358 patients met the eligibility criteria for data synthesis. Compared to the control group, the dexmedetomidine group demonstrated a significantly lower incidence of delayed graft function (OR = 0.71, 95% CI 0.52–0.97, *p* = 0.03, GRADE: Very low, I^2^ = 0%). Dexmedetomidine also significantly prolonged time to initiation of rescue analgesia (MD = 6.73, 95% CI 2.32–11.14, *p* = 0.003, GRADE: Very low, I^2^ = 93%) and reduced overall morphine consumption after renal transplant (MD = -5.43, 95% CI -7.95 to -2.91, *p* < 0.0001, GRADE: Very low, I^2^ = 0%). The dexmedetomidine group exhibited a significant decrease in heart rate (MD = -8.15, 95% CI -11.45 to -4.86, *p* < 0.00001, GRADE: Very low, I^2^ = 84%) and mean arterial pressure compared to the control group (MD = -6.66, 95% CI -11.27 to -2.04, *p* = 0.005, GRADE: Very low, I^2^ = 87%).

**Conclusions:**

This meta-analysis suggests that dexmedetomidine may potentially reduce the incidence of delayed graft function and offers a superior analgesia profile as compared to control in adults undergoing renal transplants. However, the high degree of heterogeneity and inadequate sample size underscore the need for future adequately powered trials to confirm these findings.

## Introduction

In 2021, more than 144,302 kidney transplantations were performed worldwide.^1^ Kidney transplantation is one of the most cost-effective treatments with better quality of life for patients with kidney failure,^2^ but it comes with the risk of postoperative complications, namely delayed graft function, acute rejection, and infection resulting in a poor prognosis.^3^ The prevalence of delayed graft function ranged from 20–50% in kidney transplantation in deceased kidney transplant recipients.^4^ It is generally regarded as one of the leading risk factors for chronic allograft dysfunction.^5^ Recently, there has been growing interest among anesthesiologists regarding dexmedetomidine, a non-opioid anesthetic drug, to mitigate the complications of renal transplant.

Dexmedetomidine is a highly selective α-2 agonist, which is increasingly used in all types of surgeries as an adjuvant anesthetic agent to achieve sedation, analgesic, and anxiolytic effects. It also inhibits the sympathetic tone with fewer cardiovascular effects as compared to other agents such as barbiturates or propofol.^6^ Dexmedetomidine is believed to exert good renoprotective effects by reducing ischemia-reperfusion injury via the inhibition of the JAK/STAT signaling pathways,^7^ resulting in lower incidence of delayed graft function.^8^ It can also inhibit C-fibers and Aα-fibers^9^ which reduce the perception of pain among patients and minimize the total consumption of morphine postoperatively with longer time taken to request for rescue analgesia. One study has shown that patients randomized to dexmedetomidine were associated with lower heart rate and mean arterial blood pressure by exerting its sympatholytic effect and stimulating vasodilation through alpha-2 adrenoreceptors in endothelial cells.^10^ Several published studies demonstrated that dexmedetomidine was associated with lower incidence of delayed graft function,^3^^,^^11^ lower total morphine consumption after surgery,^12^^,^^13^ longer time taken for rescue analgesia,^12^^,^^13^ while providing sedation as a non-opioid agent without respiratory depression in adult surgical patients.^6^ However, several recent published RCTs reported conflicting findings on the efficacy and safety profile of perioperative dexmedetomidine administration in patients undergoing renal transplant surgery.^14^^,^^15^ Thus, a comprehensive meta-analysis is warranted to summarise the current evidence on the use of dexmedetomidine in renal transplant patients.

We postulated that dexmedetomidine could reduce the occurrence of delayed graft function. The primary objective was to investigate the impact of dexmedetomidine on the incidence of delayed graft function in renal transplant surgery. Secondary outcomes encompassed the duration until the initiation of analgesia, overall morphine consumption following renal transplant, heart rate and mean arterial pressure variations, postoperative Visual Analogue Scale (VAS) score, frequency of acute graft rejection, post-transplant serum creatinine levels, estimated Glomerular Filtration Rate (eGFR), and urine output.

## Methods

This systematic review was conducted according to the Cochrane Handbook of Systematic Reviews of Intervention.^16^ The study protocol was published on PROSPERO, CRD42023404161 prior to the commencement of literature search.

### Sources of information and strategy

The following databases were used in the systematic search for relevant articles: MEDLINE, EMBASE, and CENTRAL (Cochrane Register of Trials). The searches were carried out systematically from its inception until March 10th, 2023. Clinical Trials.gov was searched to identify any ongoing studies. In addition, any unpublished studies found within the major databases were included. Search terms and strategy are listed in the Supplementary Table 2. Eligibility criteria were illustrated: 1) Randomised controlled trials (RCTs) or observational studies; 2) Dexmedetomidine; 3) Renal transplant or kidney recipient, 4) Adult patients, regardless of reported outcomes.

No restrictions in regard to the length of follow-up period or the language of publication were applied. Trials published as letters to editors, case series, case reports and conference abstracts were excluded. Trials comparing dexmedetomidine and controls in animal studies were excluded. References of all included studies were searched for relevant articles that fulfilled our inclusion criteria. For incomplete data, the authors of relevant studies were contacted at least three times. Review questions were formulated based on the Population (adult patients older than 18 years old undergoing renal transplant), Intervention (dexmedetomidine), Comparison (control) and Outcomes (PICO) framework.

### Outcomes of the study

The primary outcome was the incidence of delayed graft function. The definition of delayed graft function was shown in Supplementary Table 1. Secondary outcomes included the time until the initiation of analgesia, overall morphine consumption following renal transplant, heart rate and mean arterial pressure variations, postoperative Visual Analogue Scale (VAS) score, frequency of acute graft rejection, post-transplant serum creatinine levels, estimated glomerular filtration rate (eGFR), and urine output.

### Study selection and data extraction

The review was reported based on the Preferred Reporting Items for Systematic Reviews and Meta-Analyses Statement (PRISMA) 2020.^17^ Two screeners (WT, WL) were briefed by the third author (KN) on the inclusion and exclusion criteria. Titles and abstracts were selected for eligibility criteria by two authors (WT, WL). The final selection of all the included studies were decided among all three authors (KN, WT, WL). Clinical characteristics of included studies were documented independently by both authors (WT, WL). Any conflicts were solved by the third author (KN).

### Risk of bias assessment

The included studies were assessed for risk of bias with the Cochrane Collaboration Risk of Bias Assessment Tool and the Newcastle Ottawa Scale by both authors (WT, WL).^18^ Any disagreements were discussed by all authors (WT, WL, KT) until an agreement was reached. Summary of findings and assessment of the level of evidence were conducted by both authors (WT, WL) using the GRADEpro/GDT software.^19^

### Summary measures and synthesis of results

Statistical meta-analysis was performed using Review Manager version 5.4.^20^ The cut-off for statistical significance of two-tailed *p-*values was less than 0.05. Regarding dichotomous results, Odd Ratios (OR) and 95% Confidence Intervals (95% CI) were reported. For continuous outcomes, Mean Difference (MD) and 95% Confidence Interval (95% CI) were calculated. Heterogeneity of the pooled results was assessed using the I-square (I^2^) test, with values of < 40%, 40% to 60%, and > 60% denoting low, moderate, and high heterogeneity, respectively. A fixed-effect model was used to summarize the estimates of primary and secondary outcomes. If there was significant heterogeneity (I^2^ > 60%), a random-effect model was applied. When values were reported as median or interquartile range, they were converted to mean and standard deviation.^21^ Sensitivity analysis and subgroup analysis were conducted on the primary outcome.

## Results

**Figure 1:**
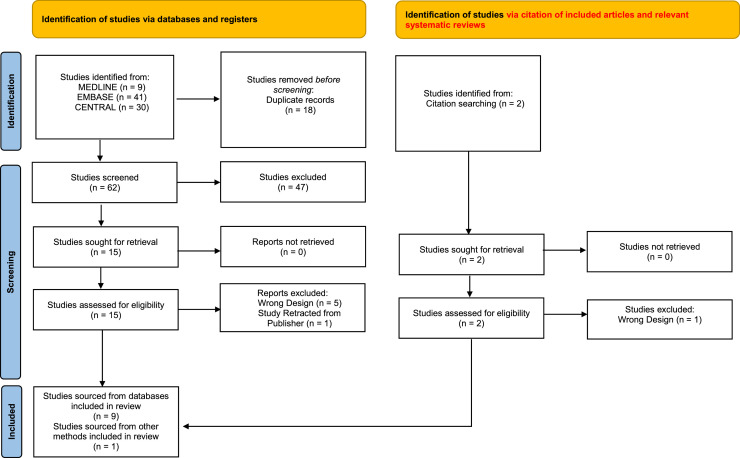
Prisma Diagram of Systematic Review. A total of 10 studies were included.

The study selection process is illustrated in the PRISMA diagram (Figure 1). Our search from databases, citations of included articles, and relevant systematic reviews generated 64 non-duplicated articles for title and abstract screening. A total of 17 articles were retrieved for full-text screening. After applying inclusion and exclusion criteria, 7 articles were excluded. The list of excluded articles is shown in Supplementary Table 3. A total of 10 studies (n = 1,358) were included in this review. Searching of trial registries found 5 ongoing studies, 1 completed but unpublished study, and 3 studies with unknown status of progress (Supplementary Table 4).

The clinical characteristics of all included studies are shown in Table 1. Of all, seven studies provided dexmedetomidine infusion^3^^,^^11^^,^^13^, ^14^, ^15^^,^^22^^,^^23^ whereas three studies administered dexmedetomidine via a bolus followed by infusion of dexmedetomidine.^12^^,^^24^^,^^25^ In terms of the timing of administering dexmedetomidine, 3 studies delivered dexmedetomidine before starting surgery^22^^,^^24^^,^^25^ whereas the rest of the studies administered dexmedetomidine within the intraoperative and postoperative period.^3^^,^^11^, ^12^, ^13^, ^14^, ^15^^,^^23^ All included studies examined the effects of dexmedetomidine on kidney transplant recipients.^3^^,^^11^, ^12^, ^13^, ^14^, ^15^^,^^22^, ^23^, ^24^ The mean age of the control group ranged from 25.45 to 65.80 years whereas the mean age of the dexmedetomidine group was 24.56 to 65.50 years. The mean body mass index of the control and dexmedetomidine groups were 20.9 to 32.2 kg.m^-2^ and 20.5 to 32.1 kg.m^-2^, respectively. Data analysis of the primary and secondary outcomes is demonstrated in Table 2. A list of summary findings and level of evidence is shown in Table 3.

**Table 1:** Characteristics and preoperative data of patients receiving dexmedetomidine or control drug. Values are mean ± standard deviation.

Author	Year	Design	Dosage of Dexmedetomidine	Type of control drug	Dosage of control drug	Method of intravenous administration	Timing of administration	Type of population	Type of kidney transplant	Mean age (Control)	Mean age (Dex)	Mean BMI (Control)	Mean BMI (Dex)	n
Negi	2014	RCT	0.5 μg.kg^−1^	Fentanyl	1.0 μg.kg^−1^	Bolus and Continuous Infusion	During Surgery	Recipient	Living	35.80 ± 10.35	34.33 ± 9.77	‒	‒	60
Wei	2015	RCT	0.6 μg.kg^−1^ before anesthesia induction, followed by 0.2 μg.kg^−1^.h^−1^ for 1 hour	Normal Saline	0.6 μg.kg^−1^ before anesthesia induction, followed by 0.2 μg.kg^−1^.h^−1^ for 1 hour	Bolus and Continuous Infusion	Before and During Surgery	Recipient	Living	‒	‒	22.00	21.20	40
Mesbah	2018	RCT	1.0 μg.kg^−1^.h^−1^	Normal Saline	1.0 μg.kg^−1^.h^−1^	Continuous Infusion	During Surgery	Recipient	Living	39.40	45.80	‒	‒	30
Chen	2020	Observational Study	0.24-0.60 μg.kg^−1^.h^−1^	Did not receive any drug	‒	Continuous Infusion	During Surgery	Recipient	Cadaveric	52.5±13.30	51.90 ± 13.60	27.60 ± 4.60	27.40 ± 4.70	780
Wang	2020	RCT	0.1–0.7 μg.kg^−1^.h^−1^	Did not receive any drug (proceeded with conventional management with fluid supplementation)	‒	Continuous Infusion	During and After Surgery (2 hours after surgery)	Recipient	Living	43 (34–53)^a^	48 [27–56]1	‒	‒	60
Yang	2020	RCT	1.0 μg.kg^−1^	Ropivacaine and Morphine PCA	Ultrasound-guided unilateral TAP block with 30 mL of 0.33% ropivacaine	Continuous Infusion	During and After Surgery (1 hour after surgery)	Recipient	Cadaveric	41.60 ± 7.60	38.50 ± 6.70	23.80 ± 3.70	23.10 ± 3.60	38
Chopade	2022	RCT	0.2 μg.kg^−1^.h^−1^	Normal Saline	0.90%	Continuous Infusion	Before Surgery (10 mins before induction of anesthesia)	Recipient	‒	41.12 ± 13.22	41.64 ± 11.15	‒	‒	50
Liu	2022	RCT	0.6 μg.kg^−1^ before anesthesia induction followed by an infusion rate of 0.4 μg.kg^−1^.h^−1^ until 30 min after surgery	Normal Saline	0.9% over 15 min	Bolus and Continuous Infusion	Before, During and After Surgery (30 min after kidney reperfusion)	Recipient	Cadaveric	42.59 ± 9.49	40.76 ± 8.78	23.20 ± 3.07	24.15 ± 3.87	65
Park	2022	RCT	0.4 μg.kg^−1^.h^−1^	Normal Saline	0.90%	Continuous Infusion	During Surgery	Recipient	Living	48 [40‒57]1**Error! Bookmark not defined.**	51 [44‒55]1	‒	‒	104
Shan	2022	RCT	0.4 μg.kg^−1^.h^−1^ (perioperative)	Normal Saline	0.4 μg.kg^−1^.h^−1^ (perioperative)	Continuous Infusion	During and After Surgery (24 hours after surgery)	Recipient	Cadaveric	43.30 ± 10.90	43.50 ± 10.7	21.10 ± 3.20	21.80 ± 3.20	111
			0.1 μg.kg^−1^.h^−1^ (postoperative for 24 hours)		0.1 μg.kg^−1^.h^−1^ (postoperative for 24 hours)									

a Median (Interquartile Range). Dex, Dexmedetomidine; BMI, Body Mass Index; n, Total sample size; RCT, Randomized Controlled Trial; PCA, Patient Controlled Analgesia.

**Table 2:** Data analysis of primary and secondary outcomes. Values are Mean Difference (MD)/Odds Ratio (OR) and 95% Confidence Interval.

Outcomes	Trial	n	I^2^ (%)	MD/OR (95% CI)	*p*-value
**Incidence of delayed graft** f**unction (**m**ain** a**nalysis)**	4	1,059	0	0.71 [0.52, 0.97]	0.030
**Incidence of** d**elayed** g**raft function**					
RCT only	3	279	0	0.51 [0.25, 1.021]	0.060
Observational study only	1	780	N/A	0.78 [0.55, 1.10]	0.160
Deceased donor	3	956	0	0.71 [0.52, 0.97]	0.030
Living donor	1	103	N/A	0.98 [0.06, 16.11]	0.990
**Duration until initiation of** r**escue analgesia**	2	98	93	6.73 [2.32, 11.14]	0.003
**Overall morphine consumption following renal transplantation**	2	98	0	−5.43 [−7.95, −2.91]	<0.001
**Heart rate**	11	600	84	−8.15 [−11.45, −4.86]	<0.001
After tracheal intubation	3	155	0	−6.71 [−7.21, −6.22]	<0.001
1 hour after induction	2	115	93	−11.23 [−27.00, 4.55]	0.160
2 hours after induction	2	110	93	−9.38 [−25.26, 6.49]	0.250
Immediately after surgery	2	110	59	−5.66 [−12.64, 1.33]	0.620
2 hours after surgery	2	110	86	−12.26 [−23.33, −1.20]	0.030
**Mean Arterial Pressure**	11	595	87	−6.66 [−11.27, −2.04]	0.005
After tracheal intubation	3	155	0	−12.00 [−12.22, −11.79]	<0.001
1 hour after induction	2	110	43	2.49 [−3.94, 8.91]	0.450
2 hours after induction	2	110	90	−7.67 [−24.42, 9.09]	0.370
Immediately after surgery	2	110	90	−7.29 [−23.56, 8.98]	0.380
2 hours after surgery	2	110	53	−3.73 [−11.67, 4.21]	0.360
**Postoperative VAS score**	9	503	90	−0.20 [−0.67, 0.26]	0.400
2 hours after surgery	2	98	94	0.24 [−1.52, 2.00]	0.790
6 hours after surgery	2	98	92	−0.61 [−1.78, 0.57]	0.310
12 hours after surgery	2	98	0	−0.17 [−0.38, 0.04]	0.120
24 hours after surgery	3	209	6	−0.49 [−0.78, −0.19]	0.001
**Post-transplant serum creatinine**	9	2,792	18	−0.03 [−0.14, 0.08]	0.620
24 hours	2	98	86	−0.33 [−1.69, 1.04]	0.640
7 days	3	941	0	−0.03 [−0.21, 0.16]	0.770
30 days	2	884	0	−0.12 [−0.31, 0.07]	0.220
90 days	2	869	0	0.00 [−0.14, 0.14]	1.000
**Post-transplant eGFR**	5	1,779	86	−0.89 [−2.21, 0.42]	0.180
30 days	3	948	45	−0.88 [−3.52, 1.75]	0.510
90 days	2	831	0	−0.50 [−0.69, −0.30]	<0.001
**Post-transplant urine output (24 hours after surgery)**	3	138	0	79.72 [−21.33, 180.76]	0.120
**Frequency of acute rejection**	3	994	0	1.33 [0.73, 2.40]	0.350

N/A, Not Applicable.

**Table 3:** Level of evidence for all primary and secondary outcomes. The level of evidence ranged from very low to moderate.

N° of trials	Risk of bias	Inconsistency	Indirectness	Imprecision	Other considerations	Absolute effect		Absolute effect	Relative Effect	Certainty
						Events/ Intervention	Events/ Control	95% CI	OR (95% CI)	
4	Very serious^a^	Not serious	Not serious	Not serious	Publication bias strongly suspected^b^	77/455 (16.9%)	137/604 (22.7%)	**54 fewer per 1,000** (from 94 fewer to 5 fewer)	**OR 0.71** (0.52 to 0.97)	⨁○○○ Very low
2	Not serious	Serious^c^	Not serious	Serious^d^	Publication bias strongly suspected^b^	49	49	MD **6.73 higher** (2.32 higher to 11.14 higher)	‒	⨁○○○ Very low
2	Not serious	Serious^c^	Not serious	Serious^d^	Publication bias strongly suspected^b^	49	49	MD **5.43 lower** (7.95 lower to 2.91 lower)	‒	⨁○○○ Very low
11	Serious^e^	Not serious	Not serious	Serious^d^	Publication bias strongly suspected^b^	338	342	MD **8.15 lower** (11.45 lower to 4.86 lower)	‒	⨁○○○ Very low
11	Serious^e^	Not serious	Not serious	Serious^d^	Publication bias strongly suspected^b^	338	337	MD **6.66 lower** (11.27 lower to 2.04 lower)	‒	⨁○○○ Very low
3	Not serious	Not serious	Not serious	Not serious	Publication bias strongly suspected^b^	23/423 (5.4%)	32/571 (5.6%)	**17 more per 1,000** (from 15 fewer to 69 more)	‒	⨁⨁⨁○ Moderate

CI, Confidence Interval; MD, Mean Difference; OR, Odds Ratio.

Explanations:

a Observational study is one of the included studies.

b Small studies with positive results.

c High degree of heterogeneity.

d Sample size of each group < 400.

e Majority of the trials possess unclear risk of bias.

Based on the overall risk of assessment, most studies were evaluated as low risk of bias, except for 4 studies that were assessed as unclear^14^^,^^22^^,^^24^^,^^25^ and 2 studies assessed as high risk of bias^11^^,^^23^ due to lack of randomization and lack of blinding of outcome assessors (Supplementary Table 5).

Of all, the definitions of delayed graft function were standardized across the included trials^3^^,^^11^^,^^15^^,^^25^ whereby delayed graft function was defined as the need for dialysis within 7 days after the kidney transplantation procedure was carried out.

### Primary outcome: incidence of delayed graft function

**Figure 2:**
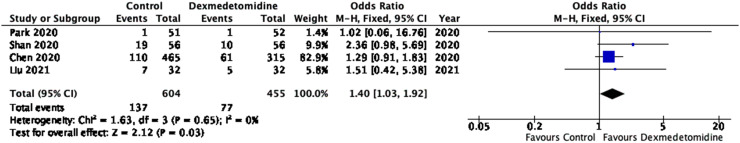
Incidence of Delayed Graft Function after surgery in patients receiving dexmedetomidine or control drugs. Dexmedetomidine significantly reduced the incidence of delayed graft function in comparison to the control group.

Based on the combined data of 4 studies (n = 1,059), the dexmedetomidine group was associated with lower incidence of delayed graft function in comparison with control (OR = 0.71, 95% CI 0.52–0.97, *p* = 0.03, I^2^ = 0%) (Figure 2).^3^^,^^11^^,^^15^^,^^25^ The level of evidence is low due to its risk of bias and strong publication bias.

However, subgroup analysis of randomized controlled trials (OR = 0.51, 95% CI 0.25–1.02, *p* = 0.06)^3^^,^^11^^,^^15^^,^^25^ and observational studies showed no significant differences between the dexmedetomidine and control groups in the incidence of delayed graft function (OR = 0.78, 95% CI 0.55–1.10, *p* = 0.16).^11^ Further subgroup investigations on deceased donor kidney transplant procedures highlighted that those patients randomly assigned to the dexmedetomidine group had a lower incidence of delayed graft function (OR = 0.71, 95% CI 0.52–0.97, *p* = 0.03).^3^^,^^11^^,^^25^

Secondary outcomes: duration until the initiation of analgesia, overall morphine consumption following renal transplant, heart rate and mean arterial pressure variations, postoperative Visual Analogue Scale (VAS) score, frequency of acute graft rejection, post-transplant serum creatinine levels, estimated Glomerular Filtration Rate (EGFR), and urine output

Two studies (98 patients) demonstrated longer duration until the initiation of analgesia in the dexmedetomidine group (MD = 6.73, 95% CI 2.32–11.14, *p* = 0.003, I^2^ = 93%) (Supplementary Fig. 3).^12^^,^^13^ Our pooled estimates also found lower overall morphine consumption following renal transplant in patients receiving dexmedetomidine (MD = -5.43, 95% CI -7.95 to -2.91, *p* < 0.0001, I^2^ = 0%) (Supplementary Fig. 4).^12^^,^^13^ The certainty of evidence is very low for both measured outcomes owing to inconsistency, imprecision and publication bias.

Our pooled data demonstrated that the dexmedetomidine group was associated with significantly lower heart rate as compared to the control group (MD = -8.15, 95% CI -11.45 to -4.86, *p* < 0.00001, I^2^ = 84%) (Supplementary Fig. 5).^22^, ^23^, ^24^, ^25^ Dexmedetomidine statistically reduced mean arterial pressure in comparison to the control group (MD = -6.66, 95% CI -11.27 to -2.04, *p* = 0.005, I^2^ = 87%) (Supplementary Fig. 6).^22^, ^23^, ^24^, ^25^ The quality of evidence was rated as very low in nature for these hemodynamic outcomes due to risk of bias, imprecision, and publication bias.

Regarding other secondary outcomes, the results of the meta-analysis are presented in Table 2.

## Discussion

This meta-analysis analyzed the impact of dexmedetomidine among patients undergoing renal transplant. In this meta-analysis, dexmedetomidine likely lowered the incidence of delayed graft function, prolonged the duration of the initiation of rescue analgesia, reduced morphine consumption, heart rate, and mean arterial pressure. As the demand for kidney transplantation is increasing worldwide, it is important to accelerate recovery and survival of the transplanted kidney, which can be achieved by reducing the incidence of delayed graft function.^26^

According to the Comprehensive Clinical Nephrology, delayed graft function is defined as the need to undergo dialysis within one week after kidney transplant.^27^ It is believed that ischemia-reperfusion injury is one of the main risk factors resulting in the delayed of graft function.^26^^,^^28^ Ischemia-reperfusion injury involves systemic vasoconstriction leading to hypoxia of renal epithelial tubular cells and endothelial cells, followed by the release of reactive oxygen species during reperfusion, the accumulation of neutrophils and the release of lytic enzymes that ultimately cause renal cell death.^26^ Gu et al demonstrated that dexmedetomidine achieves its anti-inflammatory effects by reducing Interleukin-6 (IL-6), Tumor Necrosis Factor alpha (TNF-alpha) and the Box-1 nuclear protein of the High Mobility Group (HMGB1) in animal studies.^8^

An interesting aspect that emerged from the subgroup analysis of cadaveric kidney transplants is that there was a statistically significant lower incidence of delayed graft function in the dexmedetomidine group. This may be explained by the fact that the deceased donor kidney remains longer in cold ischemia,^29^ which is defined as the period of cold perfusion of the kidney to the beginning of vascular anastomosis.^30^ The anti-inflammatory action of dexmedetomidine is believed to reduce ischemic damage, which could further reduce the incidence of delayed graft function. In keeping with previous studies,^31^ there are also other factors, namely longer donor warm ischemia time which is defined as the duration between the clamping of arteries and the beginning of the infusion of cold fluid,^30^ morbidities of the kidney recipient, nephrotoxic analgesics used perioperatively, higher body mass index of the kidney donor, and ABO incompatibilities, that could influence the incidence of delayed graft function.^32^

Dexmedetomidine is responsible for activating α-2 adrenoreceptors at the presynaptic terminals of the locus coeruleus, the dorsal horn of the spinal cord and the peripheral nervous system, which in turn inhibits the release of substance P and norepinephrine through a negative feedback mechanism,^33^ consequently terminating the transmission of C-fibers and Aα-fibers pain signals.^9^ Inhibition of neuronal firing at both the supraspinal, spinal and peripheral sites^33^ is believed to help prolong the duration until the initiation of analgesia and reduce the overall morphine consumption following renal transplant, as validated in our study.

The postoperative VAS pain score is accepted as an effective numerical tool for gauging pain intensity;^34^ however, the present review did not show significant differences in the VAS pain score between the dexmedetomidine and control groups. These contradictory results could be due to the inclusion of a limited number of RCTs with a small sample size and a substantial degree of heterogeneity in our studies, which may introduce variances. When given the same type of pain stimuli, the VAS score can differ among patients depending on their pain threshold and perception of subjective pain experiences;^35^ thus indicating lack of objectivity in the pain assessment. The substantial heterogeneity appreciated for this measured outcome could be attributed to the varied type of control drugs administered. For example, Negi et al administered 1.0 μg.kg^−1^ fentanyl infusion as a control drug whereas Yang et al used intravenous morphine as their control drug, which may introduce variances to our findings.^12^^,^^13^ Fentanyl may exert strong analgesic effects^36^ in the control group which may make the interpretation of the true effects of dexmedetomidine on postoperative pain difficult. Due to the high degree of heterogeneity across all the included studies, the pooled results need to be interpreted with caution.

In terms of its effect on the cardiovascular system, dexmedetomidine evokes a biphasic response: an initial increase in blood pressure due to the vasoconstriction effects of peripheral α-2B receptors; this leads to reflex bradycardia, followed by vasodilation as central α-2A subtype receptors predominate.^6^ The other suggested mechanism leading to bradycardia involves dexmedetomidine activating the alpha-2 adrenoreceptors in the heart which would block the cardioaccelerator nerve and stimulate the vagal nerve.^37^ Our subgroup analysis indicated that the dexmedetomidine group had a statistically lower heart rate after tracheal intubation and 2 hours after surgery and a significantly lower mean arterial pressure after tracheal intubation only. However, this pooled analysis failed to consider several factors, namely different infusion methods of dexmedetomidine (bolus vs. continuous), dosage of dexmedetomidine, type of anesthesia and patients’ baseline mean arterial pressure, and other comorbidities, that can affect both heart rate and mean arterial pressure.^38^, ^39^, ^40^ Although dexmedetomidine is associated with reduced heart rate and mean arterial pressure, none of the included studies reported significant cases of bradycardia and hypotension.^3^^,^^12^^,^^13^ In addition to its anti-inflammatory effects, animal studies postulated that the infusion of dexmedetomidine could inhibit cellular senescence by decreasing the number of senescent tubular cells and weakened the expression of senescence-associated markers such as p53, p16, and p21, subsequently alleviating renal ischemia-reperfusion injury.^41^

Acute rejection occurs due to the production of inflammatory mediators by the recipient immune cells in the transplant organ.^42^ Dexmedetomidine inhibits the release of cortisol from the adrenal glands via its inhibitory effect on the hypothalamic-pituitary axis and inhibits the release of epinephrine and norepinephrine by acting on the locus coeruleus of the sympathetic nervous system.^43^ As a result, the inflammatory responses initiated by a series of cytokines would be attenuated, as shown by a meta-analysis by Wang et al comprising 67 studies (4,842 patients) that revealed a decrease in the concentrations of pro-inflammatory cytokines such as IL-6, TNF-α, IL-1β, IL-8 and an increase in anti-inflammatory cytokines such as IL-10 after the administration of dexmedetomidine to adult patients.^43^ In contrast, our pooled results reported no significant differences in the frequency of acute graft rejection between the dexmedetomidine and control groups. The detection of acute rejection was standardized and was proven by obtaining the biopsy of the kidney allograft.^3^^,^^11^^,^^15^ Hence, the difference in results could be potentially due to several reasons: types and dosage of postoperative immunosuppressant treatments deployed across various centers, immunological risk profile of kidney transplant recipients^44^ and donor and recipient characteristics such as age, race, and gender.^45^, ^46^, ^47^

In terms of renal function, Loomba et al summarised that dexmedetomidine increases creatinine clearance, lowers Neutrophil Gelatinase-Associated Lipoprotein (NGAL) levels, and increases urinary output.^48^ These results differed from our pooled analysis as no significant differences were reported between the dexmedetomidine and control groups on post-transplant eGFR, serum creatinine, and urine output after surgery. Future RCTs that focus on the effects of dexmedetomidine on renal function under relatively more homogeneous conditions should be warranted to gain a clearer understanding of its renal effects.

In summary, dexmedetomidine was associated with a lower incidence of delayed graft function, an extended duration before requesting rescue analgesia, reduced total morphine consumption, as well as lower mean arterial pressure and heart rate in renal transplant patients. Currently, a low level of evidence and a high degree of heterogeneity limit the formulation of robust recommendations regarding the administration of dexmedetomidine in renal transplant patients.

## Declaration of competing interest

The authors declare no conflicts of interest.

## Data Availability

The authors confirm that the data supporting the findings of this study are available within the article and its supplementary materials.

## Appendix Supplementary materials

Image, application 1
