# Supplementary material for: Effects of perioperative dexmedetomidine on delayed graft function following renal transplant: a systematic review and meta-analysis
Source: Braz J Anesthesiol. 2024 Jul 2;74(6):844534. doi: 10.1016/j.bjane.2024.844534 (PMC11293508; doi:10.1016/j.bjane.2024.844534)

**BJAN-D-23-00397_Supplementary Material**

**Supplementary Table 1** Definitions of delayed graft function.

| **No** | **Author** | **Year** | **Definition of delayed graft function** |
| --- | --- | --- | --- |
| 1 | Chen | 2020 | The need for dialysis in the 7-days after transplantation |
| 2 | Shan | 2021 | The need for dialysis in the first posttransplant week |
| 3 | Liu | 2021 | The need for dialysis in the ﬁrst week after transplantation |
| 4 | Park | 2020 | The need for dialysis within the ﬁrst week after kidney transplantation |

**Supplementary Table 2** Search strategy.

| **Search Strategy for EMBASE databases (1974 to March 2023)** | | |  |
| --- | --- | --- | --- |
| *Steps* | *Search String* | |  |
| 1 | Dexmedetomidine.mp. Or exp dexmedetomidine / 17536 | |  |
| 2 | Precedex.mp. Or exp dexmedetomidine / 17078 | |  |
| 3 | Renal transplant.mp. Or exp kidney graft / 76953 | |  |
| 4 | Kidney transplant.mp. Or exp kidney graft / 73248 | |  |
| 5 | Renal donor.mp. Or exp kidney donor / 13733 | |  |
| 6 | Exp kidney graft/ or exp kidney transplantation/ or kidney recipient.mp / 176226 | |  |
| 7 | 1 or 2/ 17537 | |  |
| 8 | 3 or 4 or 5 or 6 / 186366 | |  |
| 9 | 7 and 8 / 43 | |  |
| 10 | Limit 9 to human/ 41 | |  |
| **Search Strategy for MEDLINE databases (1946 to March 2023)** | | |  |
| *Steps* | *Search String* | |  |
| 1 | Dexmedetomidine.mp. Or exp dexmedetomidine / 8455 | |  |
| 2 | Precedex.mp. Or exp dexmedetomidine / 5160 | |  |
| 3 | Renal transplant.mp. Or exp kidney graft / 108902 | |  |
| 4 | Kidney transplant.mp. Or exp kidney graft / 109954 | |  |
| 5 | Renal donor.mp. Or exp kidney donor / 174 | |  |
| 6 | Exp kidney graft/ or exp kidney transplantation/ or kidney recipient.mp / 104265 | |  |
| 7 | 1 or 2 / 8458 | |  |
| 8 | 3 or 4 or 5 or 6 / 114189 | |  |
| 9 | 7 and 8 / 10 | |  |
| 10 | Limit 9 to human / 9 | |  |
| **Search Strategy for CENTRAL databases (1946 to March 2023)** | | | |
| *Steps* | | *Search String* | |
| 1 | | Dexmedetomidine.mp. Or exp dexmedetomidine | |
| 2 | | Renal transplant.mp. Or exp kidney graft/ | |
| 3 | | 1 and 2 / 30 | |

**Supplementary Table 3** Characteristics of ongoing studies.

| **Author** | **Location** | **Status** | **Recruitment Start Date** | **Estimated completion date** | **Title** | **Comparator** | **n** | **Clinical Trial Number** |
| --- | --- | --- | --- | --- | --- | --- | --- | --- |
| Feng | China | Recruiting | 2021-11-15 | ‒ | Effects of dexmedetomidine pretreatment and posttreatment on renal function during Living Relative Renal Transplantation | Dexmedetomidine Pre-treatment and Posttreatment vs. Normal Saline | 150 | ChicTR2100045246 |
| Wang | China | Unknown | 2018-11-10 | ‒ | Application of dexmedetomidine in the anesthesia of renal transplantation | Dexmedetomidine vs. Normal Saline | 40 | ChiCTR1800019433 |
| Xu | China | Unknown | 2017-05-01 | ‒ | Effects of Dexmedetomidine on Postoperative Renal Function Recovery After Kidney Transplantation in Adult Patients | Dexmedetomidine vs. Normal Saline | 100 | NCT02874378 |
| Mehrad | Iran | Recruiting | 2017-11-06 | ‒ | Investigating the effects of dexmedetomidine infusion during surgery on renal function parameters in patients receiving kidney transplantation | Dexmedetomidine vs. Control | 50 | IRCT2017101331487N5 |
| Park | Korea | Recruiting | 2017-10-31 | ‒ | Effect of Dexmedetomidine on Renal Function and Delayed Graft Function After Kidney Transplantation | Dexmedetomidine vs. Normal Saline | 104 | NCT03327389 |
| Zhang | Taiwan | Recruiting | 2015-05-01 | 2019-09-01 | Effects of Dexmedetomidine on Delirium After Living Donor Renal Transplantation in Adult Patients | Dexmedetomidine vs. Normal Saline | 100 | NCT02509949 |
| Yeh | Taiwan | Completed | 2016-03-14 | 2020-12-17 | Effects of Dexmedetomidine on Microcirculation of Kidney Transplant Recipient | Dexmedetomidine vs. Control | 60 | NCT02707809 |
| Wen | China | Recruiting | 2021-04-25 | ‒ | The protective effect of dexmedetomidine on renal function in living donor kidney transplantation | Dexmedetomidine Pretreatment and Posttreatment vs. Whole Continuous Treatment | 90 | ChiCTR2100045802 |
| Park | Korea | Unknown | 2017-10-31 | 2019-01-15 | Effect of Dexmedetomidine on Renal Function and Delayed Graft Function After Kidney Transplantation | Dexmedetomidine vs. Control | 104 | NCT03327389 |

**Supplementary Table 4** Clinical characteristics of excluded studies.

| **Author** | **Year** | **Design** | **Reason for exclusion** | **Country** | **n** |
| --- | --- | --- | --- | --- | --- |
| Motayaghenia | 2017 | Editorial Letter | Not RCT studies | USA | ‒ |
| Singh | 2018 | RCT | Wrong Design | India | 100 |
| Amery | 2019 | ‒ | Wrong Design | USA | ‒ |
| Bai | 2021 | RCT | Wrong Design | China | 177 |
| Jiang | 2022 | RCT | Wrong Design | China | 84 |
| Dolatabadi | 2022 | RCT | Wrong Design | Iran | 40 |

**Supplementary Table 5** Risk of bias assessment of all the included studies.

| **Author** | **Year** | **Overall Risk of Bias** | **Sequence Generation** | **Allocation Concealment** | **Blinding of Participants and Personnel** | **Blinding of outcome assessment** | **Incomplete outcome data** | **Selective outcome reporting** | **Other sources of Bias** |
| --- | --- | --- | --- | --- | --- | --- | --- | --- | --- |
| Negi | 2014 | Low | Low | Low | Low | Low | Low | Low | Unclear |
| Wei | 2015 | Unclear | Low | Unclear | Unclear | Unclear | Low | Low | Low |
| Mesbah | 2018 | Unclear | Low | Unclear | Low | Low | Low | Low | Unclear |
| Singh | 2018 | Low | Low | Low | Low | Low | Low | Low | Low |
| Yang | 2020 | Low | Low | Low | Low | Low | Low | Low | Low |
| Liu | 2021 | Unclear | Low | Unclear | Low | Low | Low | Low | Unclear |
| Shan | 2021 | Low | Low | Low | Low | Low | Low | Low | Low |
| Wang | 2022 | High | Low | Low | Low | High | Low | Low | Low |
| Park | 2022 | Low | Low | Low | Low | Low | Low | Low | Low |
| Chopade | 2022 | Unclear | Low | Unclear | Low | Low | Low | Low | Unclear |
| Dong | 2022 | Low | Low | Low | Low | Low | Low | Low | Unclear |

| **Risk of Bias Assessment for Observational Study** | | | | | | | | | |
| --- | --- | --- | --- | --- | --- | --- | --- | --- | --- |
| **Study** | **Selection** | | | | **Comparability** | **Outcome** | | | **Overall** |
|  | Representativeness of the exposed cohort | Selection of the non-exposed cohort | Ascertainment of exposure | Demonstration that outcome of interest was not present at start of study | Cohorts comparable on basis of design or analysis | Assessment of outcome | Adequacy of duration of follow up | Adequacy of completeness of follow up |  |
| Chen 2020 | * | * | * | * | * | * |  |  | High risk of bias^a^ |

^a^ Scores 6/9 considered as high risk of bias.

**Supplementary Figure 1** Forest plot of delayed graft function (sub-group analysis).

**
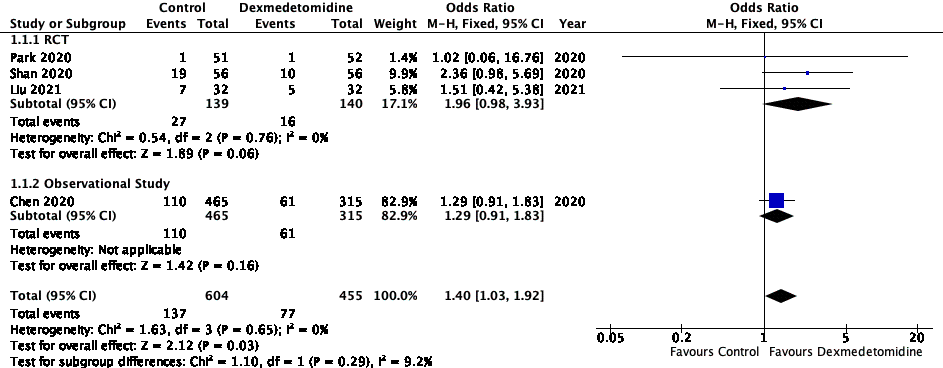
**


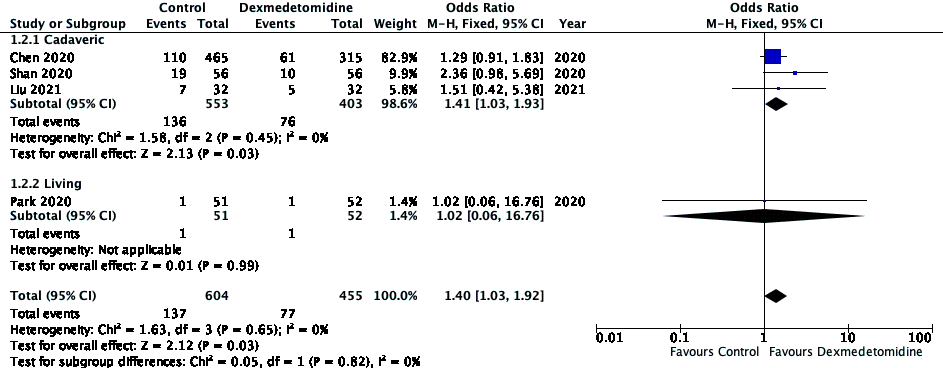


**Supplementary Figure 2** Forest plot of time taken for first analgesia.


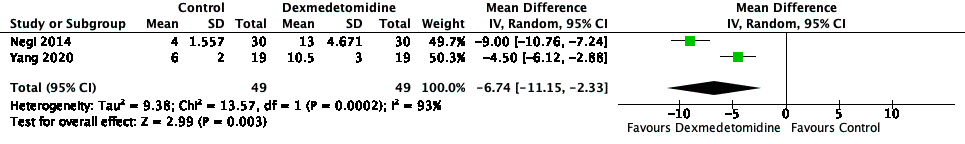


**Supplementary Figure 3** Forest plot of total morphine consumption after surgery.


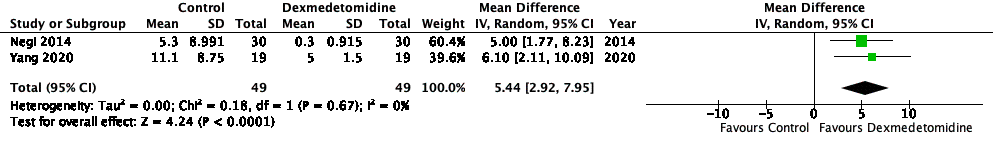


**Supplementary Figure 4** Forest plot of heart rate.


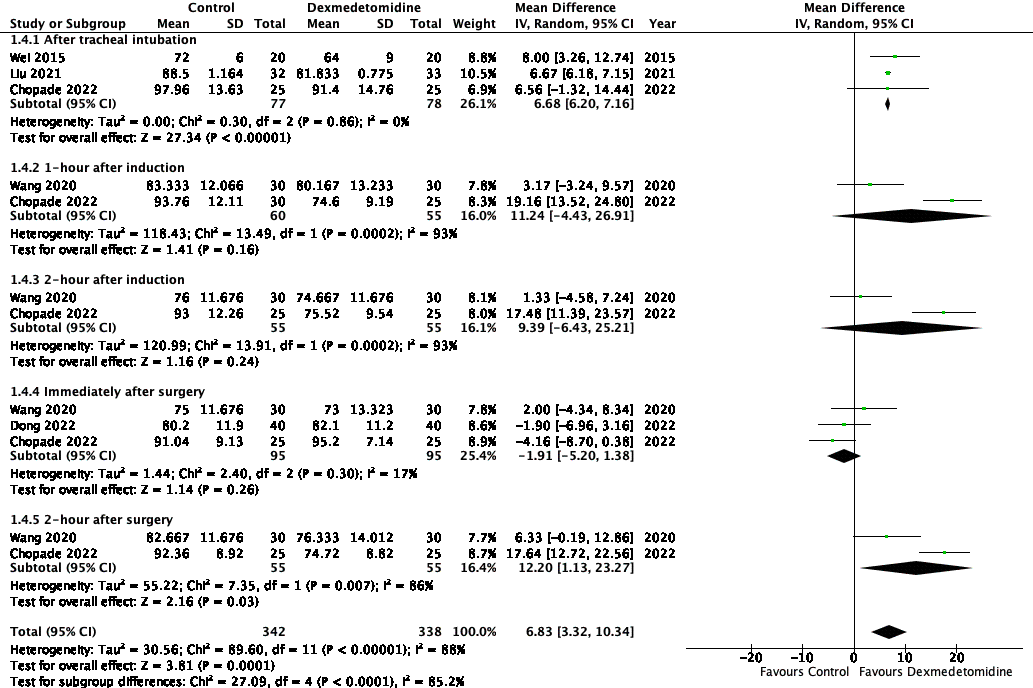


**Supplementary Figure 5** Forest plot of mean arterial pressure.


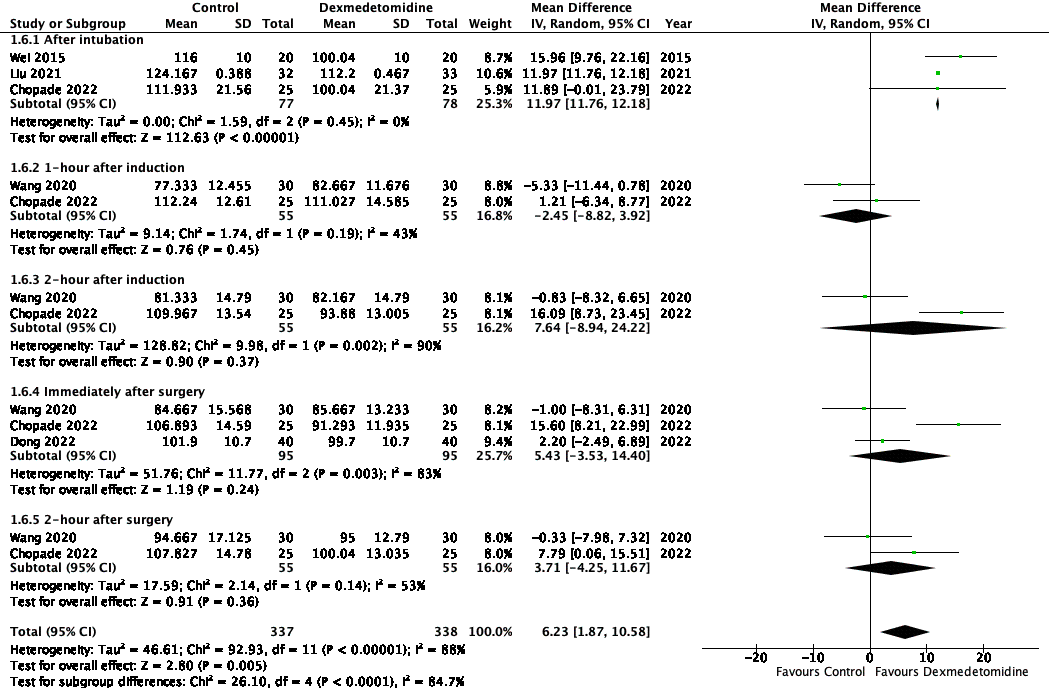


**Supplementary Figure 6** Forest plot of Visual Analogue Score (VAS)**.**


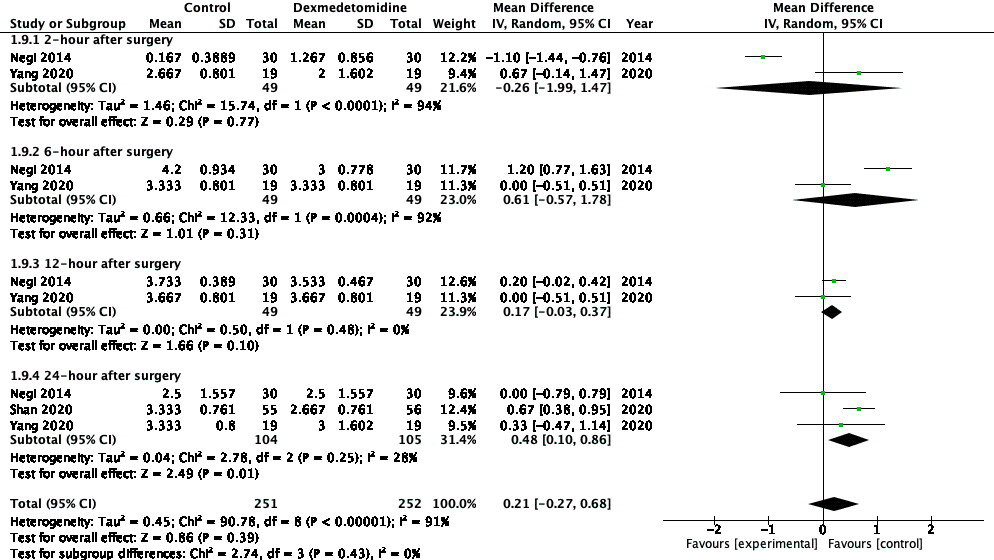


**Supplementary Figure 7** Forest plot of post-transplant serum creatinine.


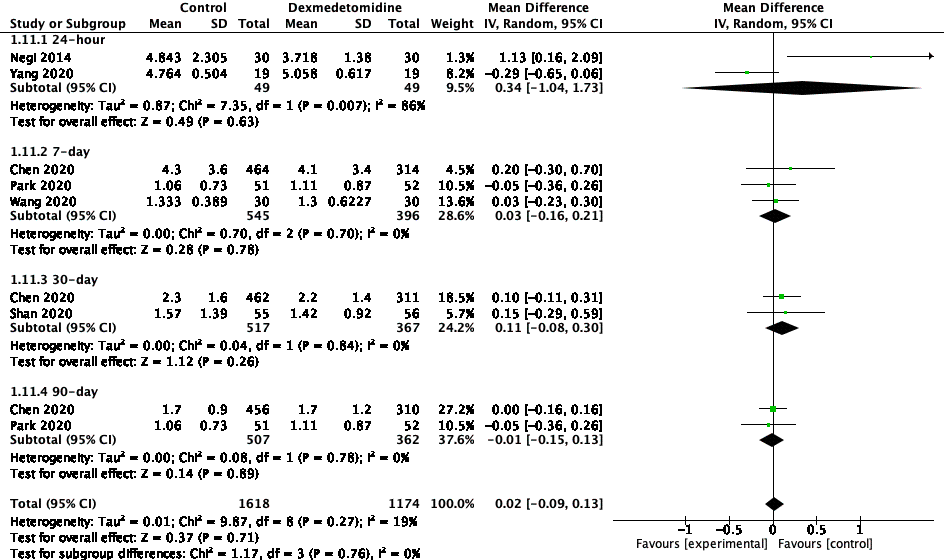


**Supplementary Figure 8** Forest plot of post-transplant eGFR.

**
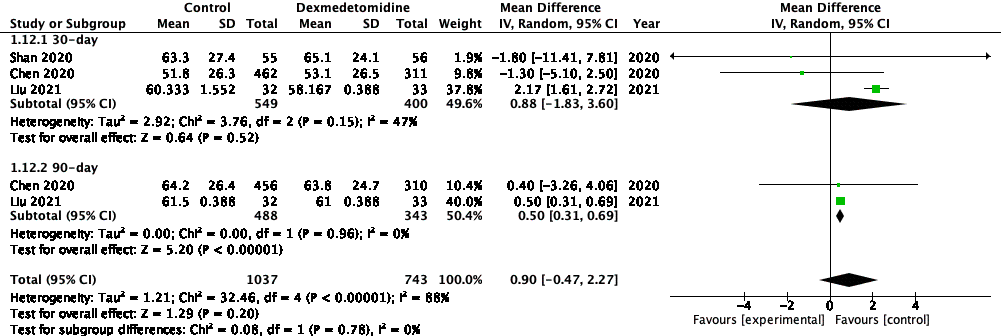
**

**Supplementary Figure 9** Forest plot of post-transplant urine output (24-hour).


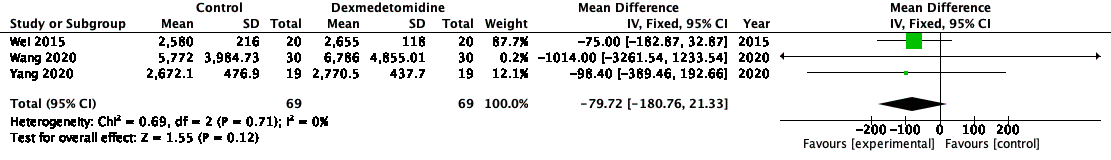


**Supplementary Figure 10** Forest plot of acute rejection.


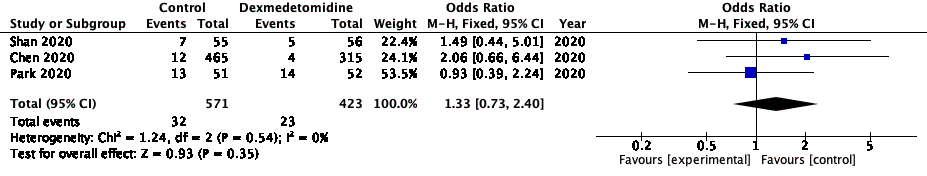

Supplement: Supplementary file 1 [file mmc1.docx]
